# Systematic stability testing of insulins as representative biopharmaceuticals using ATR FTIR-spectroscopy with focus on quality assurance

**DOI:** 10.1117/1.JBO.26.4.043007

**Published:** 2021-03-08

**Authors:** Sven Delbeck, H. Michael Heise

**Affiliations:** South-Westphalia University of Applied Sciences, Interdisciplinary Center for Life Sciences, Iserlohn, Germany

**Keywords:** infrared spectroscopy, insulin quality monitoring, protein secondary structure analysis, dry-film attenuated total reflection spectroscopy, point-of-care diagnostics

## Abstract

**Significance:** Bioactive proteins represent the most important component class in biopharmaceutical products for therapeutic applications. Their production is most often biotechnologically realized by genetically engineered microorganisms. For the quality assurance of insulins as representatives of life-saving pharmaceuticals, analytical methods are required that allow more than total protein quantification in vials or batches. Chemical and physical factors such as unstable temperatures or shear rate exposure under storage can lead to misfolding, nucleation, and subsequent fibril forming of the insulins. The assumption is valid that these processes go parallel with a decrease in bioactivity.

**Aim:** Infrared (IR) spectroscopy has been successfully utilized for secondary structure analysis in cases of protein misfolding and fibril formation.

**Approach:** A reliable method for the quantification of the secondary structure changes has been developed using insulin dry-film Fourier-transform IR spectroscopy in combination with the attenuated total reflection (ATR) technique and subsequent data analyses such as band-shift determination, spectral band deconvolution, and principal component analysis.

**Results:** A systematic study of insulin spectra was carried out on model insulin specimens, available either as original formulations or as hormones purified by ultrafiltration. Insulin specimens were stored at different temperatures, i.e., 0°C, 20°C, and 37°C, respectively, for up to three months. Weekly ATR-measurements allowed the monitoring of hormone secondary structure changes, which are supposed to be negatively correlated with insulin bioactivity.

**Conclusions:** It could be shown that IR-ATR spectroscopy offers a fast and reliable analytical method for the determination of secondary structural changes within insulin molecules, as available in pharmaceutical insulin formulations and therefore challenges internationally established measurement techniques for quality control regarding time, costs, and effort of analysis.

## 1 Introduction

For the analysis of various biopharmaceutical products including an essential approach for long-term bioactivity monitoring, systematic analytical procedures and protocols are required for determining and monitoring the molecular stability of proteins as the main active ingredients.^1^ Guidelines for stability testing of biopharmaceutical drugs are provided by the International Council of Harmonization and have been adapted by the American Food and Drug Administration (FDA), and by the European Medicines Agency. Still, new challenges for testing procedures exist, which may lead, owing to different environmental conditions as one essential factor, to different requirements for stability monitoring of biological drug substances. An innovative procedures for a reliable testing program are concerned with identification and assays for concentration determination of active substances and excipients. Furthermore, qualified and validated analytical methods for molecular stability are needed, specifying testing intervals, storage conditions (physical and chemical), and limits for bioactivity and concentrations. Compared to chemical drugs of low molecular masses, proteins from biotechnological synthesis are much more complex and therefore require extensive analytical methods for monitoring their unique stability characteristics. Stability testing procedures represent the most crucial step in specifying storage conditions of medicinal drug substances, as nowadays larger biomolecules undergo various degradation processes, depending on environmental, storage, and processing factors.^2^ These biotherapeutics are often stored as formulated solutions in glass vials, providing for highest sterility demands with the need to be kept refrigerated within a narrow temperature range during production, transportation, and shelf-life until final administration by the patients.

For insulins in commercial formulations, molecular degradation processes can be observed within their certified shelf life when these products are not stored under recommended conditions due to elevated temperatures and exposure to shear forces, which can have a significant impact on the bioactivity.^2^^–^^7^ Owing to such effects, changes in the secondary structure of the hormone occur, possibly followed by a decrease in pharmaceutical potency. These physical changes can lead to irreversible aggregation of insulin monomers via hydrophobic interactions with subsequent fibril forming. During elevated temperatures above ambient temperature, together with additional shear stress, stabilized insulin hexamers also can undergo partial misfolding and are sensitive to fibrillation. Differences between fibril dimensions are detectable, depending on whether they are formed from monomers or hexamers.^6^^,^^8^^,^^9^

International pharmacopoeia recommendations for insulin quality monitoring assays mainly rely on liquid chromatography coupled with UV detection, which is unable to distinguish between the active and inactive conformations, both possibly existing in commercial insulins after exposure to stress conditions.^10^ Infrared (IR) spectroscopy has been successfully utilized for secondary structure analysis in case of protein folding, nucleation, and subsequent fibril forming.^11^^–^^13^ An innovative method, based on infrared attenuated total reflection (IR-ATR) spectroscopy of insulin dry-films, as prepared from microliter sample volumes, is a promising analytical tool for quantifying the degree of insulin degradation, which can most likely be correlated with a decrease in biological potency as measured by classical or novel insulin assays.^14^^–^^17^ A systematic study has been carried out by us on the effect of different storage temperatures over a time period of up to three months with weekly spectroscopic measurements. By analyzing either specific shifts of the protein secondary structure sensitive absorption bands in the IR spectra or by band deconvolution, even small structural changes become detectable, especially within the spectral features calculated from second derivatives. In addition to the determination of structure-related differences, as analyzed by multivariate data analysis, a classification of different insulins, e.g., human insulin and biotechnologically produced analogs, also can be performed using a principal component analysis (PCA).

## 2 Experimental

### 2.1 Description of the Experimental Setup with Sample Preparation and Spectral Measurements

Especially samples after pharmacy delivery until use by the patient require quality control during storage under different environmental conditions. From our collection of insulin pharmaceuticals that were available from German pharmacies, examples of formulated insulin specimens, and the United States Pharmacopoeia (USP) human insulin standard (Sigma Aldrich, St. Louis, Missouri) were studied for comparison of protein structural changes. Insulin cartridges include insulin detemir (Levemir), insulin aspart (NovoRapid), NPH insulin human (Protaphane) (all produced by Novo Nordisk, Bagsværd, Denmark), insulin lispro (Humalog), NPH, insulin lispro (Humalog Mix50), insulin glargine (Abasaglar) (all produced by Eli Lilly, Utrecht, Netherlands), and insulin lispro (Sanofi, Sanofi-Aventis Groupe, Paris, France). All insulins were available as formulations in 3-ml cartridges with a biological activity of 100 international units. The abbreviation of NPH stands for Neutral Protamine Hagedorn, describing an insulin formulation with neutral pH and containing protamine as delaying substance.

Differences in the secondary structure are allegeable according to exchanges in the amino acid chain of the insulins and therefore leading to different IR absorption bands of mainly α-helical, β-sheets, random coil, or β-turn structures.^14^ These differences, and fatty acid appendages, lead to a variable duration of action within the body, with insulin categories as “fast-acting,” “intermediate-acting,” and “long-acting” for durations of around 1 to 4 h, 8 to 12 h, or 12 to 24 (48) h, and more, respectively.

**Fig. 1:**
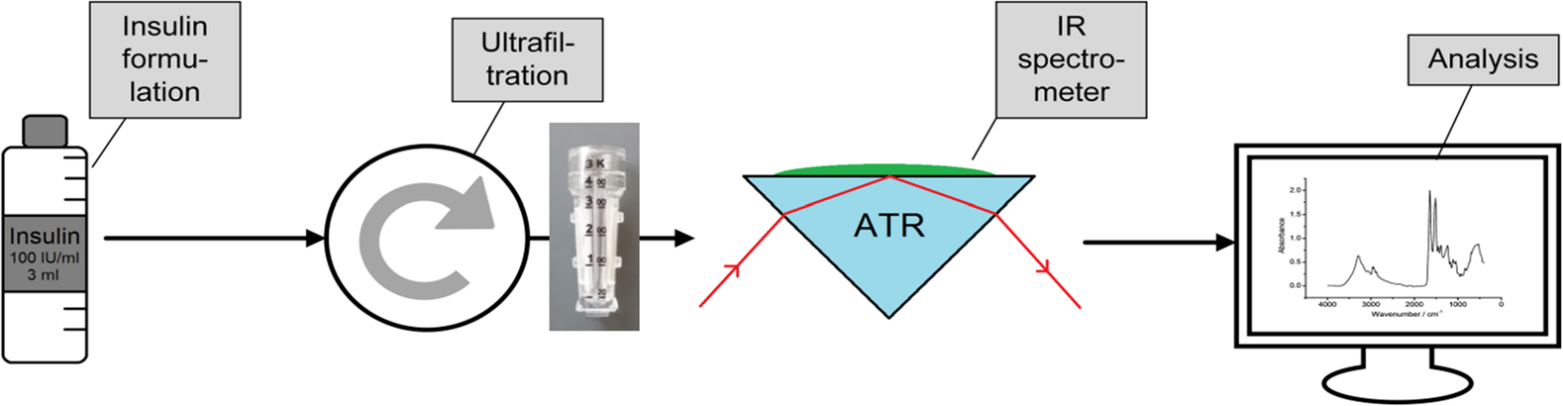
Schematics for the insulin sample treatment with ultrafiltration and subsequent IR ATR measurement including data evaluation.

In addition to the commercial insulin samples, ultrafiltrates of the original formulations were prepared using 3 kDa cutoff Vivaspin 500 centrifugal concentrators (Sartorius, Göttingen, Germany), based on vertical membrane technology for an insulin recovery of up to 90% (see also Fig. 1). The insulin formulations, aqueous solutions of insulins (purified by ultrafiltration) and the human insulin USP standard (prepared in phosphate buffer solution) were kept in 500-μl sterile sealed plastic tubes when stored in climatic exposure test cabinets at 0°C, 20°C, and 37°C, respectively, for different durations.

**Fig. 2:**
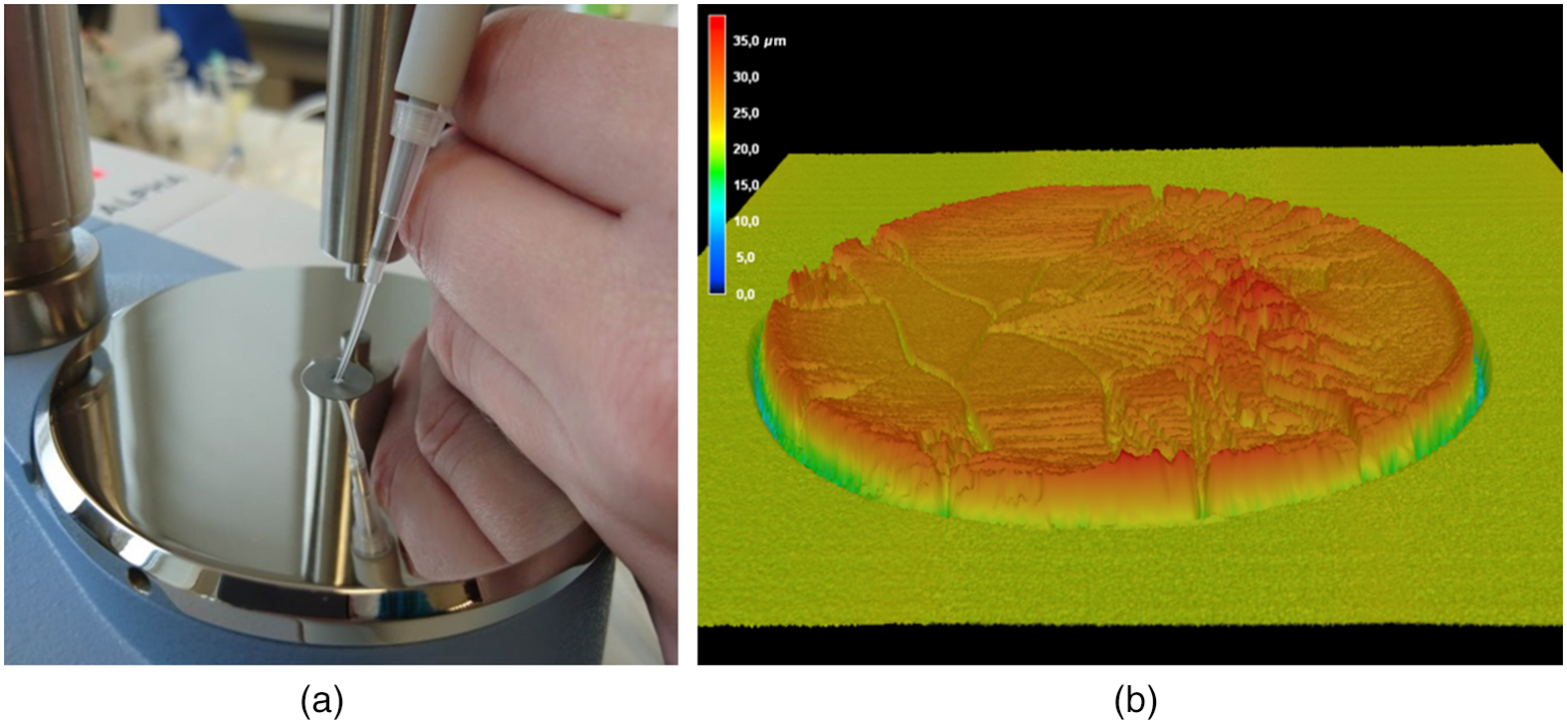
(a) Photo of the ATR accessory of a Bruker ALPHA FT-IR spectrometer with manual sample deposition (volume 1  μl) and (b) diagram of an ultrafiltrated insulin dry-film after water evaporation as measured by laser scanning.

IR spectra were recorded using a Bruker ALPHA spectrometer (Bruker Optics, Ettlingen, Germany), equipped with an ATR accessory (diamond prism with single bounce reflection). Spectral resolution of each 200 coadded interferograms was $4\;{\mathrm{cm}}^{-1}$, and for apodization a Blackman-Harris three-term function was applied including the standard Mertz method for phase correction. Fourier-transformation was performed with a zerofill as appropriate for a wavenumber spacing of about $1\;{\mathrm{cm}}^{-1}$. A temperature-stabilized DLaTGS (deuterated L-alanine doped triglycene sulphate) was used as a detector. All spectra have been recorded using the OPUS software for background, and for the sample spectra. In Fig. 2(a), a photo of the ATR accessory is shown illustrating the manual application of a sample solution volume of 1  μl using a standard Eppendorf pipette with sterile disposable tip. The sample water was evaporated by a constant air blow from a membrane pump aimed at the sample. The drying process was continuously monitored using the single beam IR spectrum from the tracing mode of the OPUS software. After 2 min, a dry film had been formed. From an aqueous solution of Levemir insulin that was purified by ultrafiltration, a representative dry film is shown in Fig. 2(b) as measured by laser scanning microscopy (model VK-X100, Keyence, Neu-Isenburg, Germany); the diagram is illustrating a slightly uneven surface with an average thickness of 7±0.4  μm after the evaporation process. Layer thickness calculations were from averaging three independent diagonal line scan measurements (VK analytics module software, Keyence, Neu-Isenburg, Germany). The solution had been deposited on a gold-sputtered microscope slide with a spread to 2 mm in diameter. Dry-films from formulated insulin specimens usually resulted in thinner films of around an average height of 2±0.1  μm due to reduced concentrations with a slight indication of a so-called coffee-ring structure.^18^ For the formulations, glycerol as part of the formulation was still present due to the lower vapor pressure compared to water.

**Fig. 3:**
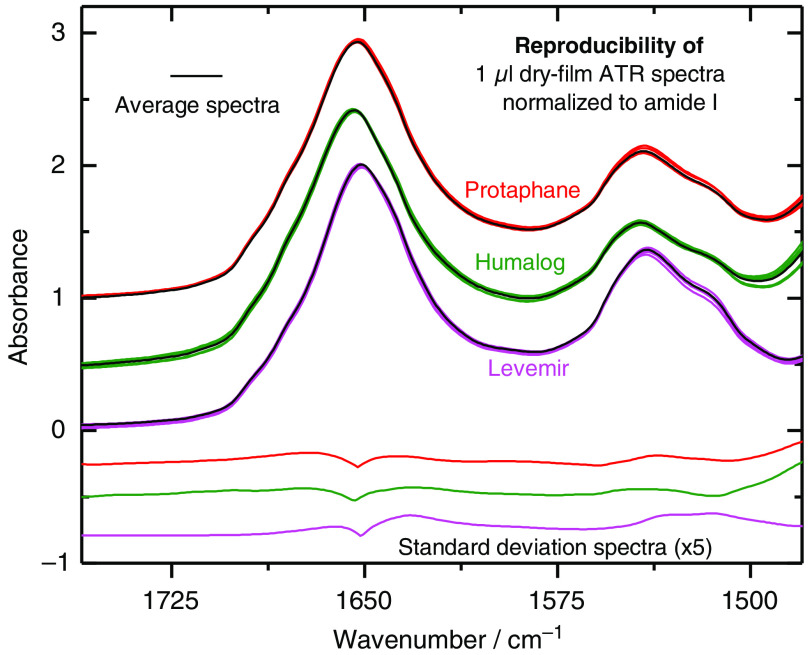
Spectra from three different insulin pharmaceuticals, demonstrating the reproducibility of dry-film preparations after minimum-maximum normalization with standard deviation spectra from five repeat measurements (offset for clarity).

For a systematic study of the reproducibility of the aforementioned dry films of three selected insulin specimens and their IR-ATR spectra, five samples from each insulin cartridge were measured (see Fig. 3). Under standardized spectroscopic conditions, dry films were produced from 1-μl samples and ATR spectra were recorded. For a better comparison of the maximum band positions, all spectra were minimum-maximum normalized within the spectral interval of 1780 to $1580\;{\mathrm{cm}}^{-1}$ (amide I band). An average spectrum was calculated for each spectral data set, and the corresponding standard deviation (SD) spectrum. According to the validation protocol of the Bruker Alpha spectrometer, the wavenumber scale precision is within a maximum shift of $0.5\;{\mathrm{cm}}^{-1}$. For the measured insulin samples (Protaphane, Humalog, and Levemir), the amide I band maxima including spread were determined at wavenumbers of $1651.34\pm 0.29\;{\mathrm{cm}}^{-1}$ (Levemir), $1654.00\pm 0.25\;{\mathrm{cm}}^{-1}$ (Humalog), and $1652.98\pm 0.41\;{\mathrm{cm}}^{-1}$ (Protaphane). Spectral differences within the amide I and II interval are noticeable and have been used for score plots calculated from a PCA; see also below in Sec. 2.2.

**Fig. 4:**
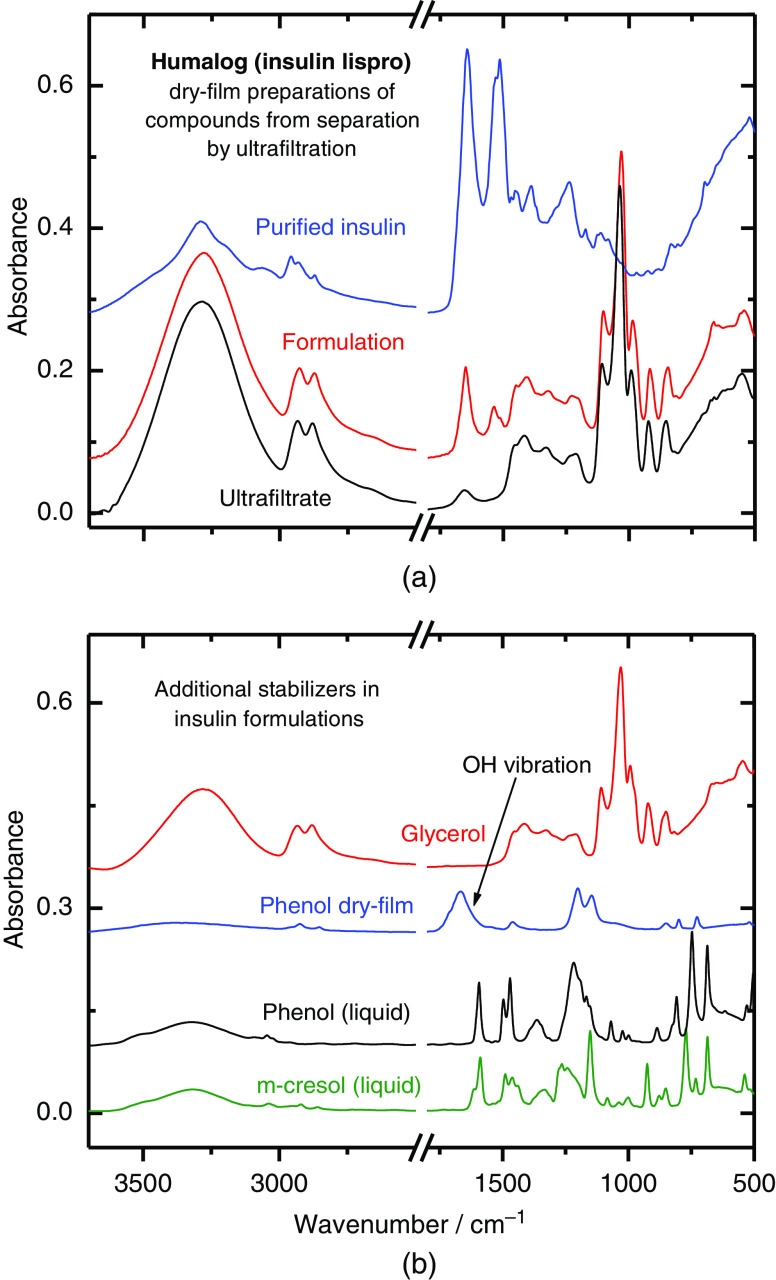
(a) ATR dry-film spectra of an insulin lispro sample as formulation, and fractions after ultrafiltration, which are represented by the filtrate and the purified insulin obtained after washing cycles and prepared as aqueous solution; (b) spectra of the different excipients, as found in the insulin formulations.

In Fig. 4(a), examples of ATR spectra are illustrated that are giving insight into the original formulation and separated fractions obtained by ultrafiltration. In this case, the formulation spectrum is dominated by absorption bands of glycerol as the major excipient left over after water evaporation. Similar spectral features are recorded from the filtrate apart from the separated insulin fraction. As shown in Fig. 4(b), other excipient compounds such as phenol or m-cresol are found at low concentrations not contributing significantly to the dry-film spectrum; the same applies to glycerol within the protein amide I and II band interval, as demonstrated earlier by us.^19^ However, still some traces of water are left over and visible from the deformation vibration band at $1650\;{\mathrm{cm}}^{-1}$.

### 2.2 Spectral Data Analysis

For analyzing the insulin secondary structure alteration, the analytical evaluation tools were either based on the monitoring of specific band shifts or a band deconvolution within the amide I and II interval, allowing also a precise quantification of secondary structure changes and providing an explanation of maximum band position shifts. Prior to band deconvolution, a spectral resolution enhancement, using second derivative spectra by means of the Savitzky–Golay algorithm with convolution by cubic polynomials with nine supporting points, was carried out. Subsequent curve fitting within the amide I region was done with Gauss–Lorentz shaped functions with final proof by comparing the resulting sum band second derivative spectrum to that of the experimental spectrum.

Further multivariate data analysis such as PCA provides not only information on structural changes, but also allows the identification of insulin subclasses by appropriate score plots.^20^ The reason is that the individual spectra are influenced by the sequence of amino acid side chains of the different insulin analogs. The significant changes within the insulin samples at various storage temperatures due to misfolding and subsequent fibrillation allow the assumption of a decrease in bioactivity. The data evaluation was carried out using the QUANT2 analysis module of the OPUS software for PCA calculations of spectral data sets, comprised of multiple-repeat spectra of different insulin pharmaceuticals.

## 3 Results

### 3.1 Identification of Different Commercial Insulin Specimens Using ATR-Spectroscopy

**Fig. 5:**
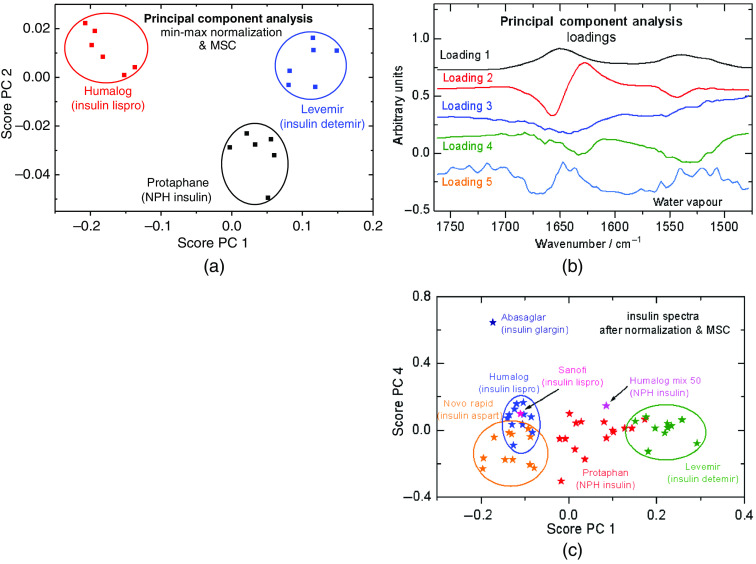
(a) Score plots from the first two principal components of repeat spectra recorded from three different classes of manufactured insulin formulation (see also text; corresponding dry-film ATR spectra have been given in Fig. 3); (b) loading spectra after the PC analysis of seven different insulins as measured during a ten-week storage at 37°C; (c) corresponding score plots from PC one and four, illustrating the possible classification of insulins.

As already shown in Fig. 3, spectra of different formulated insulin pharmaceuticals obtained by our standardized dry-film preparation method can be used for classification. In Fig. 5, score plots from different insulins are shown: Protaphane as NPH insulin, an intermediate–acting insulin, Humalog (insulin lispro, an approved regular short-acting analog insulin) and Levemir (insulin detemir as a long-acting specimen). The spectra, as obtained after a 2 min drying period (sample volumes 1  μl each taken from a previously untapped cartridge), were min-max normalized and subsequently adjusted by multiplicative scatter correction. This method has been suggested as extra spectral pre-processing for protein quantification from ATR dry-film spectra.^21^ The data were restricted to the amide I and II interval of 1760 to $1478\;{\mathrm{cm}}^{-1}$. Spectral differences are so significant that well-isolated clusters have been formed.

Another PCA analysis has been performed using four different formulated insulin samples (lispro, aspart, NPH human, and detemir) as stored for ten weeks at 37°C. In addition, three more insulin samples (glargine, NPH lispro, and lispro from another manufacturer) as measured after freshly taken from a cartridge have been included in the analysis. In Fig. 5(b), the first five loading spectra from PCA are shown, which all include characteristic spectral features of the insulin amide I and II bands.^20^ Best discrimination of the insulin classes was achieved when plotting scores of PC one and four after the aforementioned spectral pre-processing [Fig. 5(c)]. Compared to the results from Fig. 5(a), there is no explicit differentiation possible between the different insulins, but clusters are formed for representing the different insulin classes such as fast-acting (lispro and aspart), intermediate-acting (NPH) and long-acting (detemir, glargine).

### 3.2 Molecular Stability of Commercial Insulins Influenced by pH Change and Temperature

**Fig. 6:**
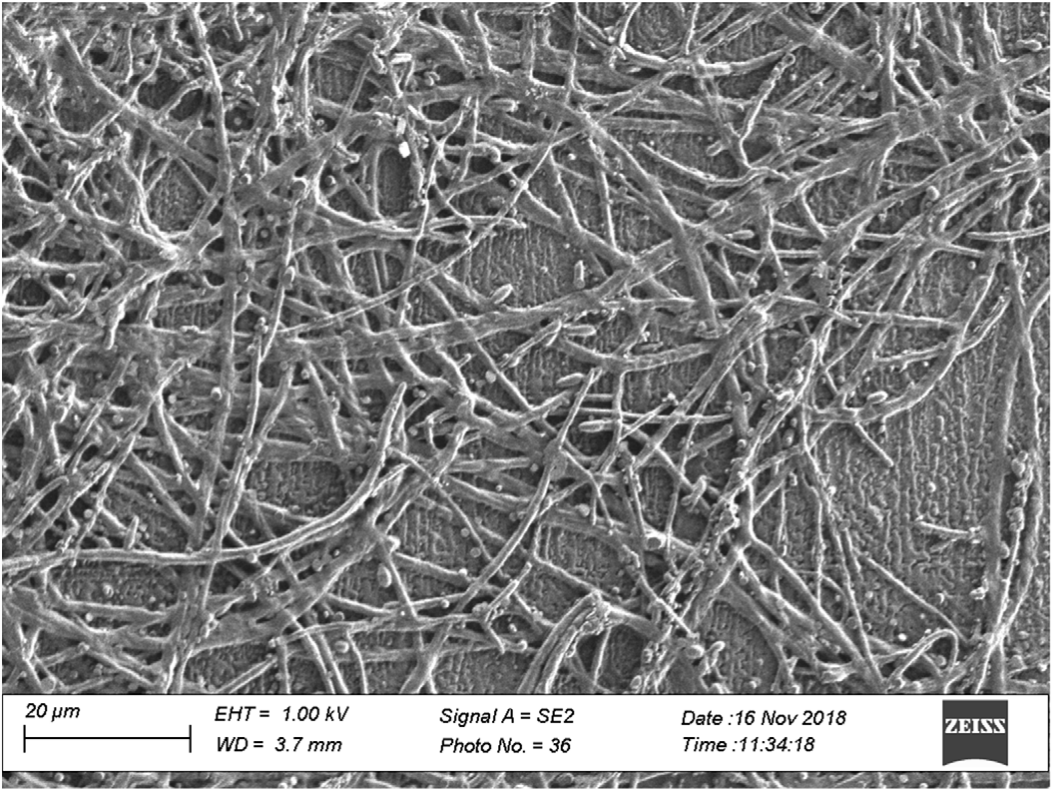
SEM image (Zeiss Sigma 300 VP, Oberkochen, Germany) of the fibril structure within an insulin detemir sample after fibrillation, measured as dry-film from a solution on a gold-sputtered microscope slide; the sample had been diluted by a factor of 10 for achieving a better optical separation of the insulin fibrils.

**Fig. 7:**
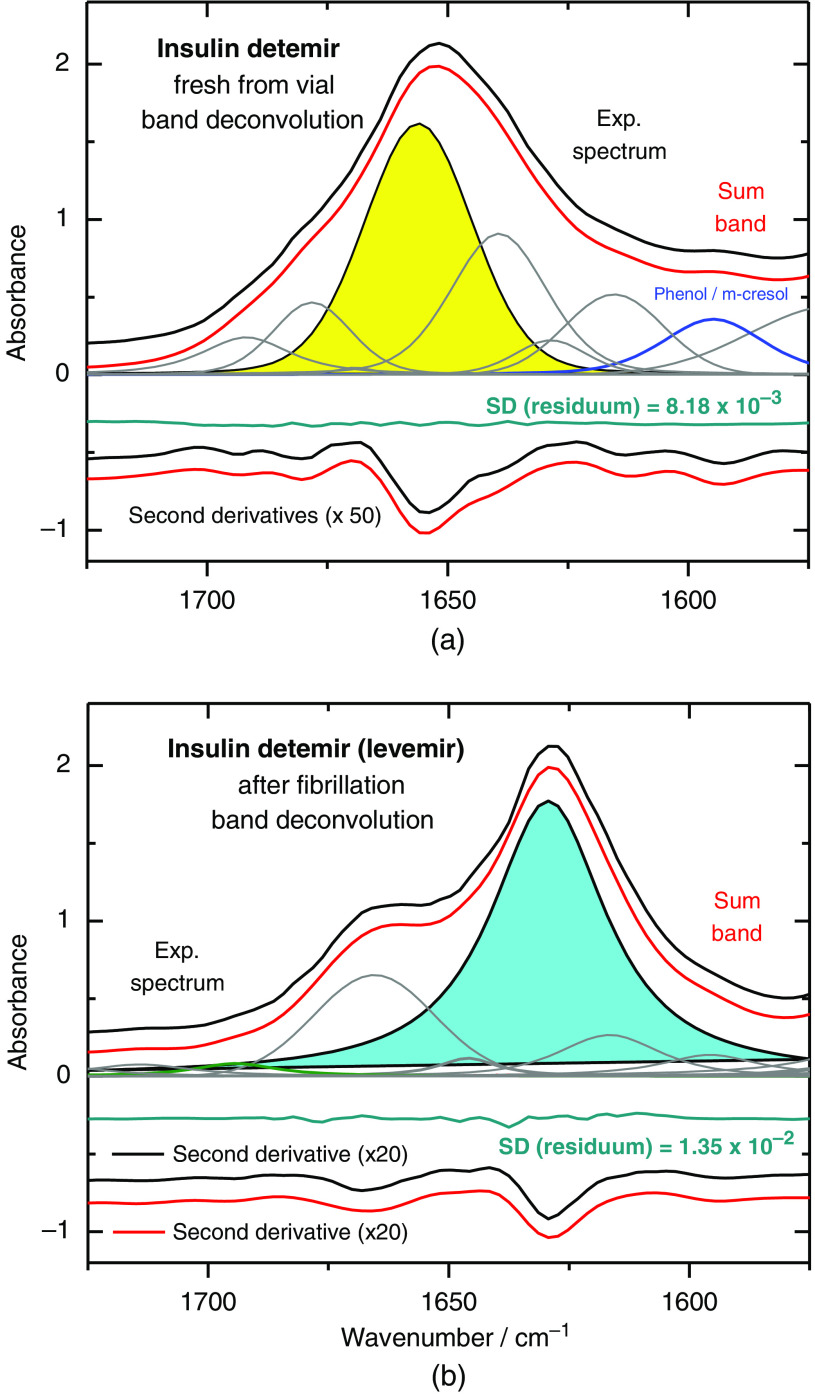
Structural analysis of the amide I band, containing information on the secondary structure components of α-helical, β-sheet, side-chain and turn conformations, and of stabilizing agents such as phenol and m-cresol (for further information on band positions of secondary sub-structures, see also Ref. 14). (a) Band positions have been selected after calculating the second derivative spectra of the reference sample of insulin detemir and (b) the sample after 48 h of incubation at 37°C in buffer solution at pH=1.5 resulting in misfolding and fibrillation.

Protein misfolding and fibril formation could be produced by changing the pH of one selected insulin detemir sample to 1.5 and storing it at 37°C for two days.^9^ In addition to the recorded and analyzed ATR-spectra, scanning electron microscopic (SEM) images have clearly shown the fibril formation (Fig. 6). Curve fitting results with spectral data between 1725 and $1575\;{\mathrm{cm}}^{-1}$ featured the secondary structure compositional shift from mostly α-helical structures (yellow) to a high percentage of β-sheet (light blue) conformation [see Figs. 7(a) and 7(b)].

As described in Sec. 2.2, the determination of the percentage distribution of the different substructure components within the amide I band (see Fig. 7) has been realized based on band fitting using OPUS software (Bruker, Ettlingen, Germany). In Fig. 7(a), the secondary structure composition of intact insulin detemir is shown, as analyzed by band deconvolution. The experimental spectrum was unfolded within the amide I band interval, offering insight into the band characteristics of α-helical, parallel and antiparallel β-sheet, side-chain and β-turn structures, as mentioned earlier.^14^ By calculation of the second derivatives, band positions can be identified from determining characteristic minima within the selected spectral interval. As main component of the bioactive insulin, the α-helical substructure with band maximum around $1655\;{\mathrm{cm}}^{-1}$ needs to be mentioned, and the weaker bands around $1695\;{\mathrm{cm}}^{-1}$ (antiparallel beta sheet) and $1630\;{\mathrm{cm}}^{-1}$ (parallel beta sheet). Compared to the active form, the spectrum of an insulin fibril sample [see Fig. 7(b)] mostly consists of band components assignable to the parallel β-sheet secondary structure and turns, as can be seen around $1670\;{\mathrm{cm}}^{-1}$. A vibrational band, visible in the active insulin sample, which can be assigned to random secondary structures ($1640\;{\mathrm{cm}}^{-1}$), is missing within the fibril spectrum. For the validation of the band deconvolution, second derivatives of both, the experimental and the sum band spectrum, were compared to each other and the SD between the normalized spectra was calculated.

### 3.3 Molecular Stability of Insulins Influenced by Temperature Stress as Analyzed by Band Shifts

**Fig. 8:**
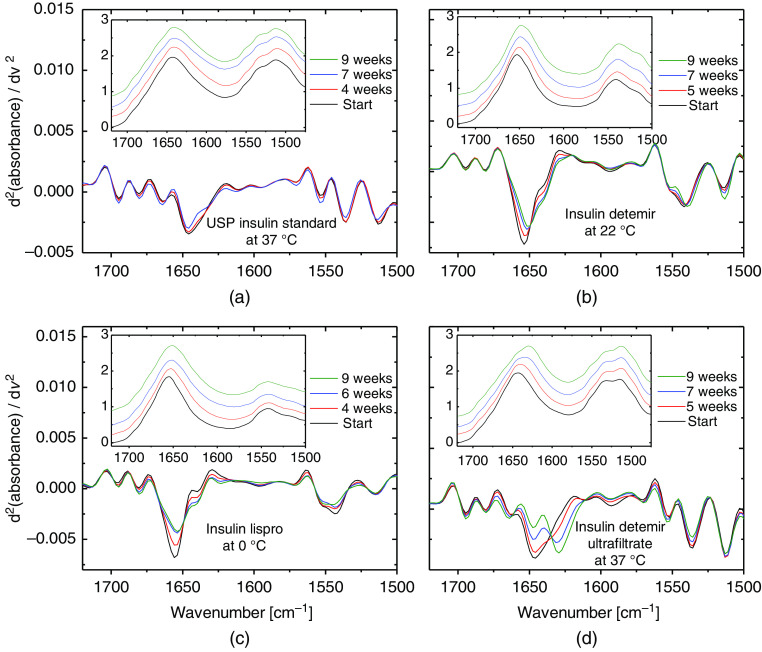
Secondary structure changes detected within the amide I and II bands, and in second derivative insulin-ATR spectra, when stored at different temperature conditions over nine weeks: (a) and (c) dry-film spectra from pure insulin solutions, and (b) and (d) from insulin formulation samples.

Focusing on the amide I and II band analysis and the related second derivative ATR spectra between 1720 and $1500\;{\mathrm{cm}}^{-1}$, changes within the secondary structure of the insulin molecules have been detected by small, but specific spectral changes in all insulin samples that were dependent on their storage conditions [see Figs. 8(a)–8(d)]. These changes correlate well with alterations in their secondary structure and will therefore influence the pharmacological activity. Especially under storage conditions at high temperature (37°C), an increased amount of β-sheet fractions could be detected by band deconvolution, underlining the protein structural misfolding [see Figs. 8(a) and 8(c)]. A validation method for the band deconvolution is provided by comparison of the second derivative spectra from both experimental and sum spectra after curve fitting.^13^

**Fig. 9:**
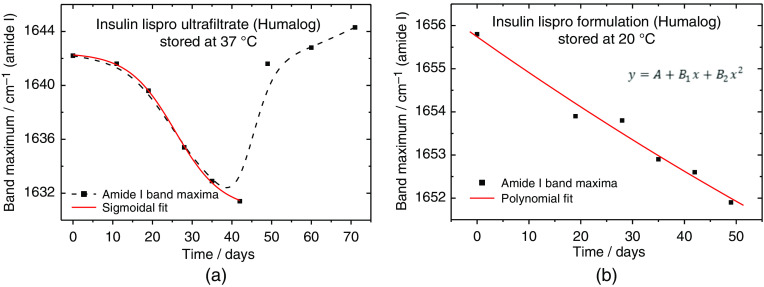
(a) Plot of the amide I band positions during long term storage of an ultrafiltrated pure insulin lispro sample at 37°C, fitted with a sigmoidal Boltzmann function; (b) same plot obtained for a humalog insulin formulation stored at 20°C.

Compared to the ultrafiltrated insulin samples, only small shifts were detected for the formulations, suggesting that the added stabilizers are protecting the insulin effectively from accelerated misfolding. This can also be seen for two representative experiments, where insulin lispro has been stored as ultrafiltrated and originally formulated sample under 37°C and 20°C, respectively (see also Fig. 9). Here, shifting of amide I band maxima was found throughout different long-term experiments with human insulin, and with its formulated or purified analogs, following a sigmoidal trend of the band shifts over time due to fibril forming kinetics.^22^ In the case of the insulin lispro ultrafiltrate, a reverse shift after 45 days could be explained by the increased formation of β-turn fractions, as these structures show band maxima around 1668 to $1681\;{\mathrm{cm}}^{-1}$, as well an increased width of the α-helical vibrational band due to changes in the physical properties within the fibril network, leading to a migration of sum band maxima towards higher wavenumbers^14^ (for further insight, see also Sec. 3.4). Nevertheless, this hypothesis needs further experimental support.

Major misfolding events on a molecular basis of the insulin molecules can be detected by analyzing the maximum amide I band positions. In general, more or less obvious changes in the secondary structure were detected within the amide I and II bands, and in second derivative insulin-ATR spectra, when stored under deviating temperature conditions from those, recommended by the manufacturers.

### 3.4 Insulin Stability Influenced by Temperature Stress as Analyzed by Band Deconvolution

**Fig. 10:**
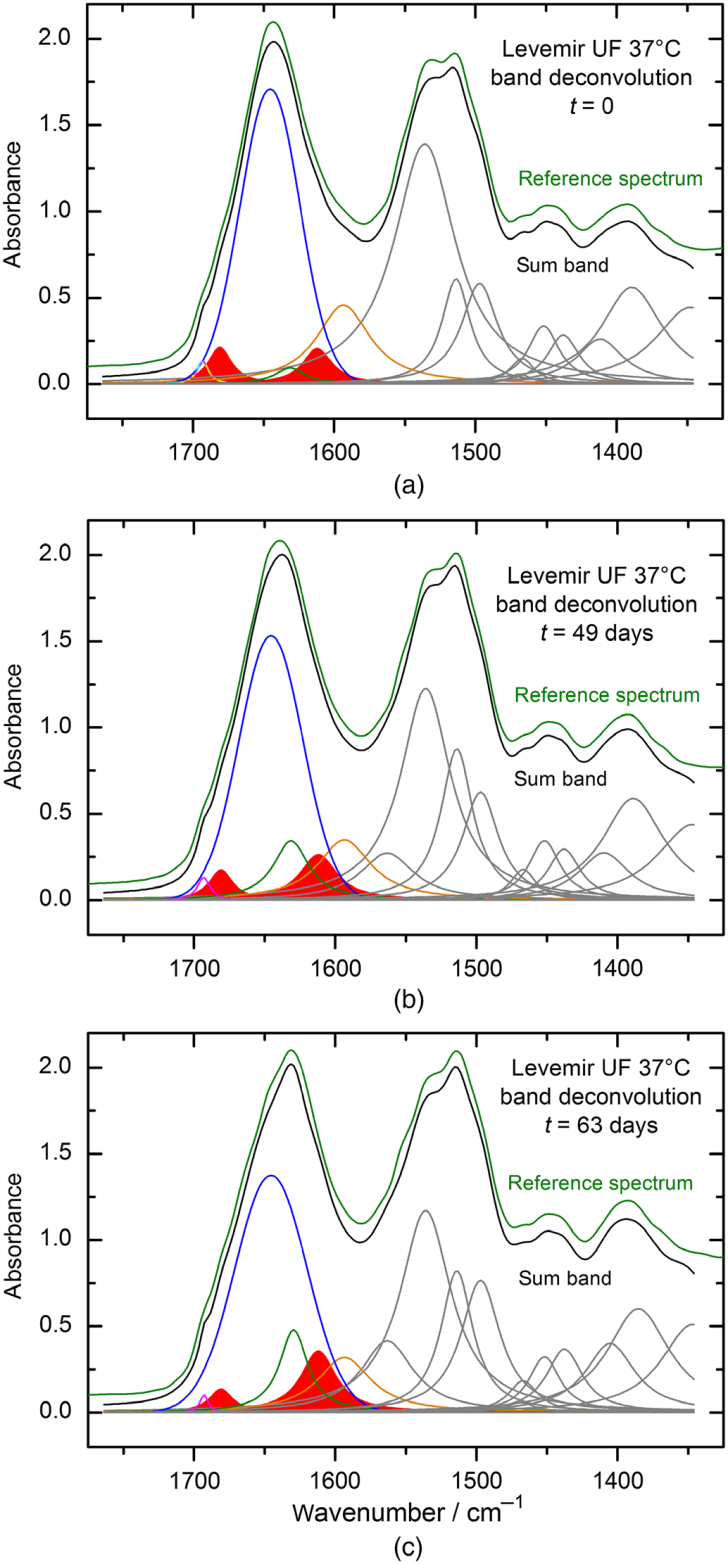
Structural analysis of pure insulin detemir bands within the interval of 1750 to $1350\;{\mathrm{cm}}^{-1}$, containing information on the secondary structure components in different subunits with a special focus on the amide I band. The subplots (a)–(c) represent the status after different time lapses of insulin solution storage at 37°C.

In Sec. 3.2, an example for band deconvolution has been given with a particular baseline set prior to band fitting. Here, the interval has been extended, which includes also the amide II band of proteins and further lower wavenumber bands. Results of the spectral deconvolution are presented for sample spectra of purified Levemir insulin as prepared in aqueous solution and stored up to nine weeks at 37°C. Dry-film ATR spectra had been obtained as described above for weekly withdrawals of μl-volume samples. In Fig. 10, the fitting results are illustrated with highlighting also the band component at $1630\;{\mathrm{cm}}^{-1}$ (red), attributable to β-sheet subunits. In addition to the individual band components, the sum band spectrum is shown with a slight offset compared to the experimental spectrum.

**Fig. 11:**
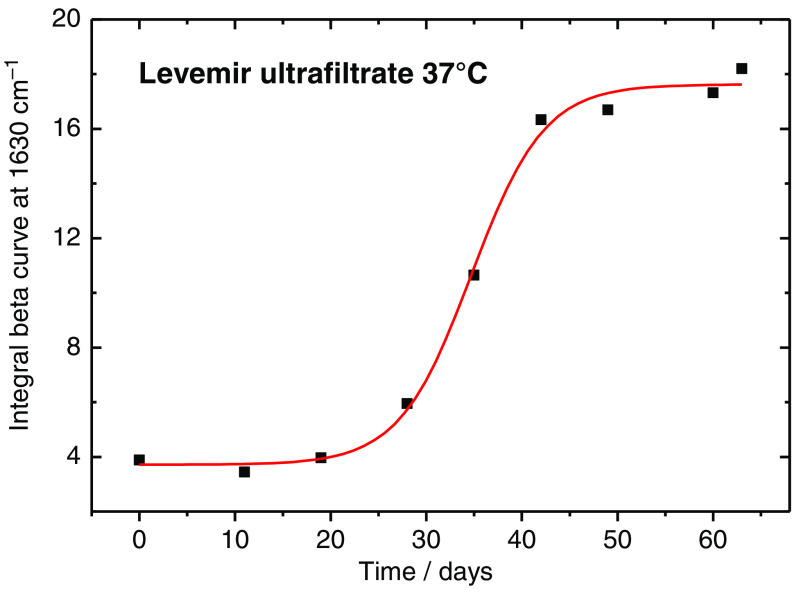
Plot of the amide I band component integral of the band centered at $1630\;{\mathrm{cm}}^{-1}$, attributable to changes in beta sheet formation during long term storage of an ultrafiltrated pure insulin Levemir sample at a temperature of 37°C and characterized by a sigmoidal function obtained by a least-squares fit.

**Fig. 12:**
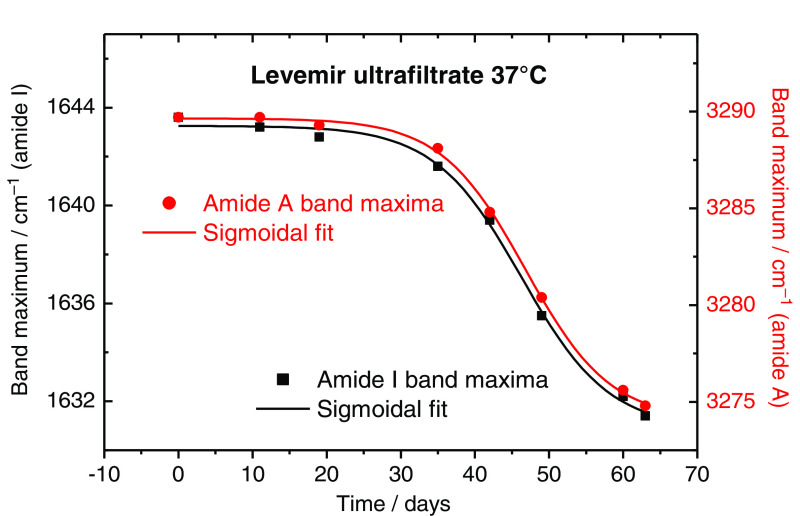
Plot of the maximum band positions derived from amide I (black curve) and Amide A (red curve) bands, using the same insulin dry-film spectra as analyzed in Fig. 11. Results from the sigmoidal least-squares fits are given in Table 1.

After analyzing each dry-film spectrum of the insulin detemir samples by applying a Levenberg–Marquardt fitting algorithm to the set of fixed band positions, followed by further manual optimization of band shapes, the integral of the $1633\;{\mathrm{cm}}^{-1}$ β-sheet band was also determined using the OPUS software. The results of the β-sheet secondary structure related band integral, as ratio of the sum of band integrals attributable to all different secondary structures, are plotted in Fig. 10. Based on the outcome of the fitting procedure from Fig. 10, a sigmoidal Boltzmann function [see Eq. (1)] can be applied for the data set, the results of which are summarized in Table 1. Regarding the misfolding, differences in the physical half-life of insulins from the sigmoidal fits for both maximum band positions derived from amide I and amide A bands (see Figs. 11 and 12) and integrals of the β-sheet band can be explained by the complex structure of the amide I band. While the band deconvolution directly provides information on the amount of α-helical and β-sheet secondary structures within the amide I band, shifts of the maximum band positions are influenced by all different substructures, and therefore do not exclusively exhibit a direct relationship with α- to β-changes. With integration of this individual band, the kinetics of the folding process can be followed, as elucidated with Figs. 11 and 12. After a time period of 25 days, the misfolding process shows its onset, which is obtained without the stabilizing formulation excipients. As mentioned above, we used the following equation for fitting the band maximum with $v_{\max}$ versus time (t); for other parameters, see Table 1:

$$v_{\max}=\frac{v_{A}-v_{E}}{1+e^{\left(t-t_{0}\right)}}+v_{E}.$$ (1)

**Table 1:** Overview of the constants for both band integral and maximum band position Boltzmann functions, and information on the physical half-life of insulin detemir misfolding and the coefficient of determination.

Constants	$1633\;{\mathrm{cm}}^{-1}$ band integral	Amide I max. band position	Amide A max. band position
$v_{A}$ (start)	3.7±0.3	$1643.3\pm 0.2\;{\mathrm{cm}}^{-1}$	$3289.7\pm 0.{1\;\mathrm{cm}}^{-1}$
$v_{E}$ (end)	17.6±0.3	$1630.9\pm 0.5\;{\mathrm{cm}}^{-1}$	$3274.1\pm 0.3\;{\mathrm{cm}}^{-1}$
$t_{0}$ (center time) with $v_{\max}=(v_{A}+v_{E})/2$	34.7±0.6 days	46.3±0.8 days	46.8±0.4 days
$R^{2}$	0.9962	0.9979	0.9994

**Fig. 13:**
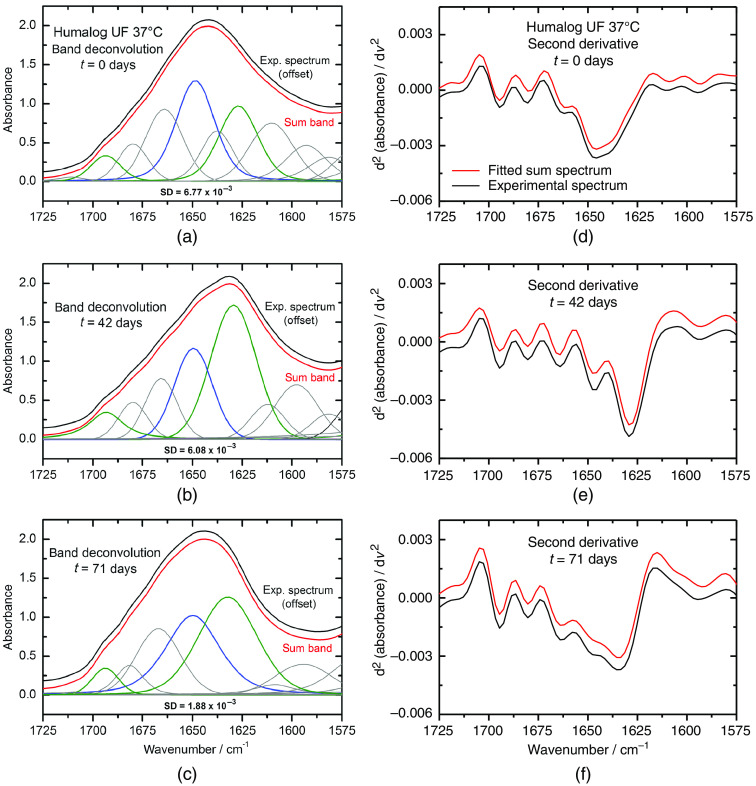
Experimental (black curves) and calculated (red curves) amide I bands of a purified insulin lispro (Humalog) sample, measured at selected storage times as dry-films on an ATR diamond from 1-μl sample volumes. (a)–(c) Secondary structure related sub bands are shown under the amide I curves, representing the determined ratio of alpha helices, beta sheets, random coils and turns; comparison of second derivative spectra as calculated from the experimental and sum band spectra are shown in the subplots on the right. Calculations have been performed using the Savitzky–Golay algorithm with nine-point cubic polynomials. (d)–(f) This final step can be used for validating the band deconvolution.

Referring to the results of the fibril forming kinetics from Fig. 9, as analyzed by maximum band shift determinations, further detailed information on the secondary structure composition of the purified insulin lispro samples was obtained via the band deconvolution described previously. In Fig. 13, the deconvoluted spectra of three dry-films in the interval of the amide I band, recorded at the beginning, after 42 days, and after 71 days are shown, respectively. The first two spectra Fig. 13(a) and Fig. 13(b) represent the start and the end of the sigmoidal function in Fig. 9, where the α-to-β transition can be observed. The third spectrum Fig. 13(c) shows the band deconvolution after ten weeks, where different changes within the amide I band can be detected. A possible explanation is given above in Sec. 3.3.

In all diagrams, experimental spectra of the 1-μl dry-film samples (black) are plotted directly above the sum band curves (red). The sum bands originate from the single bands underneath, which in turn represent the different secondary structures. Colored in blue are the parts belonging to the α-helices ($∼1650\;{\mathrm{cm}}^{-1}$) and in green are anti-parallel ($∼1695\;{\mathrm{cm}}^{-1}$) and parallel ($∼1630\;{\mathrm{cm}}^{-1}$) β-sheet fractions given. Corresponding SD between experimental spectra and sum bands, calculated by least squares, provide information on the quality of band deconvolution. Besides the quality estimate, as given by SDs, a validation method is needed for the approval of the correctness of both band position and band appearance (width, height, and form). A reasonable method is by comparison of the second derivative spectra from both experimental and sum spectra after band deconvolution. For example, the validity testing of correct band decomposition was performed using the three spectra from Figs. 13(a)–13(c), which showed very satisfying conformity between experimental and band decomposition results, as obvious from the second derivative spectra presented in Figs. 13(d)–13(f).

## 4 Discussion

There is certainly concern about quality control using analytical reference methods within the production process. Regulatory authorities such as the FDA in the United States have been established for ensuring the high quality of biopharmaceutical products with a demand for suitable assays. Most analytical methods, i.e., high-performance liquid chromatography (HPLC) with UV-detection,^23^ nuclear magnetic resonance (NMR) spectroscopy,^23^^–^^26^ or photometric assays such as by Bradford,^27^ can be used to determine total protein content, but cannot be applied for secondary structure analysis, which is most essential for assessing the insulin’s pharmacological activity.

NMR techniques using a benchtop NMR analyser have been described by Yu et al., a method that is based on the transverse relaxation rate of water protons to serve as a sensitive indicator for the detection and quantification of visible and sub-visible protein aggregates.^25^^,^^26^ This method is non-destructive as no cartridge opening is required for inspection. Another study on the aggregation of insulin analogs has been reported by Zhou et al.,^28^ who used size exclusion chromatography together with concomitant dynamic light scattering and Raman spectroscopy.

Moreover, with our global market availability of drugs, also questions about the supply chain management that may impact product quality, in particular of biopharmaceutical products with proteins and peptides as main active agents with a certified potency. Especially, for the potency determination, more complicated and elaborate assays are required. For example, glucose clamp experiments, performed in subjects with type I diabetes, could be carried out for insulin with an evaluation of the time-of-action profile under standard conditions.^29^^,^^30^^,^^31^ Glucose clamp experiments are characterized by the measurement of the glucose-lowering effect of administered insulin by a variable glucose infusion rate, so that blood glucose concentrations are maintained or clamped at a predefined target level, by which the pharmacodynamic effect of exogenous insulin can be assessed. An alternative can be animal tests as described in the US Pharmacopeia in its chapter on insulin assays for the potency assessment of insulin based on rabbits.^15^^,^^32^^,^^33^ Recently, a replacement of the US pharmacopoeia rabbit blood sugar method by an *in vitro* cell test has been suggested by us.^16^ The European Pharmacopeia does not require an *in vivo* bioidentity test, and their quality assurance is solely based on a HPLC method quantifying total insulin, but not necessarily discriminating from, e.g., misfolded fractions.

The ATR IR-spectroscopic method presented here offers a multi-purpose approach, combining the protein’s secondary structure analysis with concentration quantification and identification of insulin subclasses, respectively.^19^ Combined with the aforementioned *in vitro* cell test, which offers insight into the biopotency of selected samples, this method could replace the current section for insulin quality control in international Pharmacopoeias. In comparison to quality assurance by HPLC methods, ATR IR-spectroscopy of protein-enriched insulin dry-films can detect early stages of protein misfolding, secured by reliable validation protocols based on multivariate data analysis, as described here. An important question, which needs to be answered when establishing such assays, is the following: Due to the exponential progression of misfolding of insulin molecules, leading to fibril formation after passing through a moderate lag time with individual early misfolding events followed by nucleation, will it be still sufficient to keep the regulated 5% deviation thresholds concerning insulin quantity to guarantee highest insulin quality?

## 5 Conclusions

Samples of both formulated insulins and their ultrafiltrates were stored under different temperature conditions (0°C, 20°C, and 37°C, respectively) in 500-μl sterile sealed plastic tubes, using climatic exposure test cabinets. Same treatment was applied for the USP insulin prepared as aqueous phosphate buffer solution. Much light is shed onto the spectroscopic background, when different insulin formulations and insulins isolated from whole drug formulation are exposed to “aging” conditions for following also early protein misfolding processes. Our assay shows that insulin protein secondary structure can be monitored by IR amide I maximum band shift determinations, as well as by band deconvolution and band area integration providing quantitative figures about degradation.

Another significant advantage of the suggested IR spectroscopic assay using dry-film preparations of μl formulation sample volumes is its possible application as a point-of-care method due to being reagent-free, fast, and requiring only a low budget for instrument acquisition. Concerning complex potency assays, animal testing or clamp experiments on diabetic patients are required, representing the current established methods, but the information available from Fourier-transform IR-ATR spectroscopy could be possibly used for a replacement.
